# Dataset on positive mental health of Indonesian, Malaysian, and Thailand university students

**DOI:** 10.1016/j.dib.2020.106314

**Published:** 2020-09-14

**Authors:** Tutut Chusniyah, Jas Laile Suzana Jaafar, Apitchaya Chaiwutikornwanich, Dedi Kuswandi, Ari Firmanto, Arif Mustopa, Gebi Angelina Zahra

**Affiliations:** a State University of Malang, Indonesia; b University of Malaya, Malaysia; c Chulalongkorn University, Thailand

**Keywords:** Mental health, Subjective happiness, Forgiveness, Humility, Information literacy self-efficacy

## Abstract

The present data article provides a descriptive and analytical exploration on the links between positive mental health, subjective happiness, forgiveness, humility, and information literacy self-efficacy among 969 undergraduate students from Indonesia, Malaysia, and Thailand. There are 355 males and 614 females with an average age of 20.47 years and a standard deviation of 1.87. Respondents are recruited by simple random sampling using face to face method, at one time data retrieval during 2019. The Indonesian, Malaysian and Thailand-version questionnaires were provided to each groups of participants according to their nationality and native language, using back-to-back analysis. The socio-demographic details of the respondents, descriptive statistics, confirmatory factor analysis, correlation matrix of all variables in all groups according to country, results of regression analysis of variables, and Kruskal Wallis for all five variables in all groups are provided.

## Specifications Table

**Table:** 

Subject Area	Psychology
Specific Subject Area	Psychology (General)
Type of Data	Microsoft Excel table Extensible Stylesheet Language (.xlsx)
How Data was Acquired	Questionnaires
Data Format	Raw, Analyzed
Parameters for data collection	The variables were positive mental health, subjective happiness, forgiveness, humility, and information literacy self-efficacy.
Description of data collection	Data and questionnaires used (Malaysian, Indonesian, and Thais) are provided as supplementary material.
Data Source Location	Indonesia, Malaysia, and Thailand
Data Accessibility	With the article

## Value of the Data

• This data sets provide information on positive mental health, subjective happiness, forgiveness, humility, and information literacy self-efficacy of undergraduate students in three countries in Southeast Asia.
• Researchers will be able to use the data to determine how the five variables intertwined and correlated.
• Researchers will be able to elicit the ethnic and gender differences among the three groups.
• The data can be used to re-examine the psychometric properties of each questionaires in Southeast Asia cultural setting using Confirmatory Factor Analysis, correlation and Kruskal Wallis analysis.
• The data is resourceful to deepen cultural and international understanding on attributes of mental health among non-Western communities.

## 1 Data Description

The .csv file supplied presents the data of subjective happiness, forgiveness, positive mental health, humility, and information literacy self-efficacy of undergraduate students in State University of Malang (Indonesia), University of Malaya (Malaysia), and Chulalongkorn University (Thailand). Data was collected during 2019. We provided the Indonesian, Malaysian and Thailand-version questionnaires to each groups of participants according to their nationality and native language, using back-to-back analysis which will explained further in questionnaire section below [1]. In addition, if you want to know the basic information of the sample population and the results of descriptive statistics, please see Tables 1 and 2. Correlation among all variables was processed using Pearson bivariate correlation with the help of SPSS software. Factor loading as a result of Confirmatory Factor Analysis (CFA) of all variables can be seen on Table 3 while matrix correlation between all respondents and country described on Table 4. The statistical results for regression analysis can be seen in Table 5. Table 6 shows Kruskal Wallis results from all variables.

**Table 1:** Distribution of socio-demographic data.

Item		Frequency	
		Number	Percentage
All Subjects			
Gender	Male	355	36.64%
	Female	614	63.36%
*Total*		*969*	*100%*
			
Age	17 or less	5	0.51%
	18-20	497	52.28%
	21-23	406	41.89%
	24-26	60	6.19%
	27-29	0	0.00%
	30 or more	1	0.10%
*Total*		*969*	*100%*
Indonesia			
Gender	Male	142	42.90%
	Female	189	57.10%
*Total*		*331*	*100%*
			
Age	17 or less	3	0.91%
	18-20	239	72.21%
	21-23	87	26.28%
	24-26	1	0.30%
	27-29	0	0.0%
	30 or more	1	0.30%
*Total*		331	*100%*
Malaysia			
Gender	Male	148	46.25%
	Female	172	53.75%
*Total*		320	*100%*
			
Age	17 or less	0	0.00%
	18-20	32	10.00%
	21-23	229	71.56%
	24-26	59	18.43%
	27-29	0	0.0%
	30 or more	0	0.00%
*Total*		320	*100%*
Thailand			
Gender	Male	65	20.44%
	Female	253	79.56%
*Total*		318	*100%*
			
Age	17 or less	2	0.62%
	18-20	226	71.07%
	21-23	90	28.30%
	24-26	0	0.00%
	27-29	0	0.0%
	30 or more	0	0.00%
*Total*		318	*100%*

**Table 2:** Descriptive statistics.

	N	Maximum	Minimum	Mean	SD	Variance
*All Subjects*						
Positive Mental Health	969	45.00	9.00	33.67	5.65	31.86
Subjective Happiness	969	21.00	4.00	14.74	3.56	12.66
Forgiveness	969	95.00	19.00	67.89	11.72	137.57
Humility	969	14.00	2.00	9.38	2.68	7.10
Information Literacy Self Efficacy	969	50.00	10.00	33.66	5.61	31.50
*Indonesia*						
Positive Mental Health	331	45.00	9.00	34.52	6.35	40.30
Subjective Happiness	331	21.00	5.00	14.02	3.27	10.71
Forgiveness	331	95.00	29.00	73.85	12.19	148.64
Humility	331	14.00	6.00	10.68	2.82	3.32
Information Literacy Self Efficacy	331	50.00	10.00	35.53	7.01	49.13
*Malaysia*						
Positive Mental Health	320	45.00	13.00	33.71	5.60	31.35
Subjective Happiness	320	21.00	4.00	16.23	3.69	13.62
Forgiveness	320	77.00	19.00	59.32	6.92	47.92
Humility	320	14.00	2.00	7.87	2.77	7.68
Information Literacy Self Efficacy	320	50.00	20.00	37.75	4.31	18.62
*Thailand*						
Positive Mental Health	318	45.00	14.00	32.73	4.71	22.15
Subjective Happiness	318	21.00	5.00	13.98	3.23	10.45
Forgiveness	318	95.00	39.00	70.31	10.00	100.07
Humility	318	14.00	2.00	9.55	2.59	6.69
Information Literacy Self Efficacy	318	50.00	17.00	36.74	4.88	23.80

**Table 3:** Confirmatory factor analysis result.

No	Variable	Items	Factor Loadings								
			Malaysia			Indonesia			Thailand		
1	Positive Mental Health	1	,67			,72			,70		
		2	,66			,82			,79		
		3	,74			,72			,78		
		4	,66			,75			,54		
		5	,69			,71			,46		
		6	,72			,75			,65		
		7	,64			,60			,50		
		8	,67			,80			,54		
		9	,59			,64			,51		
2	Subjective Happiness	1	,85			,77			,59		
		2	,82			,73			,78		
		3	,65			,64			,83		
			B	S	FvsR	B	S	FvsR	B	S	FvsR
3	Forgiveness	1	,34			,83			,73		
		2	,54			,88			,72		
		3	,75			,53			,58		
		4		,64			,67			,72	
		5		,73			,83			,77	
		6		,81			,89			,76	
		7		,76			,90			,66	
		8		,54			,61			,63	
		9			,51			,67			,39
		10			,60			,82			,59
		11			,70			,75			,55
		12			,50			,83			,52
4	Humility	1	,71			,55			,62		
		2	,53			,73			,60		
5	Information Literacy Self Efficacy		Basic	Middle	High	Basic	Middle	High	Basic	Middle	High
		1	,58			,85			,72		
		2	,64			,92			,82		
		3	,57			,91			,83		
		4			,52			,65			,49
		5		,36			,84			,82	
		6		,41			,69			,70

**Table 4:** Correlation matrix of all variables for all subjects and each country.

No	Subject/Variable	1	2	3	4
	All Subject				
1	Positive Mental Health				
2	Subjective Happiness	.476⁎⁎			
3	Forgiveness	.189⁎⁎	-.051		
4	Humility	.158⁎⁎	-.217⁎⁎	.284⁎⁎	
5	Information Literacy Self Efficacy	.344⁎⁎	.251⁎⁎	-.014	-.200⁎⁎
	Indonesia				
1	Positive Mental Health				
2	Subjective Happiness	.474⁎⁎			
3	Forgiveness	.270⁎⁎	.151*		
4	Humility	-.223⁎⁎	-.132*	.117*	
5	Information Literacy Self Efficacy	.407⁎⁎	.257⁎⁎	.100	-.224⁎⁎
	Malaysia				
1	Positive Mental Health				
2	Subjective Happiness	.451⁎⁎			
3	Forgiveness	.056	.055		
4	Humility	-.227⁎⁎	-.083	-.086	
5	Information Literacy Self Efficacy	.281⁎⁎	.243⁎⁎	-.014	-.196⁎⁎
	Thailand				
1	Positive Mental Health				
2	Subjective Happiness	.620⁎⁎			
3	Forgiveness	.245⁎⁎	.155⁎⁎		
4	Humility	-.164⁎⁎	-.151⁎⁎	.179⁎⁎	
5	Information Literacy Self Efficacy	.375⁎⁎	.179⁎⁎	.101	-.055

⁎⁎ Correlation is significant at the 0.01 level (2-tailed).

⁎ Correlation is significant at the 0.05 level (2-tailed).

**Table 5:** Regression analysis of variables.

Variables	B	T	Sig
(constant)	9.733		.000
Subjective Happiness	.654	14.927	.000
Forgiveness	.115	8.690	.000
Humility	-.192	-3.212	.001
Information Literacy Self Efficacy	.227	8.818	.000
			
F	120.325		.000
Adjusted R Square	.330

**Table 6:** Kruskal Wallis result of all subject.

Variables	Country	N	Mean Rank	df	Sig
Positive Mental Health	Indonesia	331	533.40	2	.000
	Malaysia	320	493.04		
	Thailand	318	426.54		
Subjective Happiness	Malaysia	320	619.23	2	.000
	Thailand	318	419.21		
	Indonesia	331	418.43		
Forgiveness	Indonesia	331	630.37	2	.000
	Thailand	318	551.55		
	Malaysia	320	268.51		
Humility	Indonesia	331	621.47	2	.000
	Thailand	318	496.02		
	Malaysia	320	332.88		
Information Literacy Self Efficacy	Malaysia	320	544.61	2	.000
	Thailand	318	483.27		
	Indonesia	320	429.02		

## 2 Design, Materials, and Methods

### 2.1 Participants characteristic

The present data article aims to investigate the impact of subjective happiness, forgiveness, humility, and information literacy self-efficacy on mental health among undergraduate students from Malaysia, Indonesia, and Thailand, as well as to determine the difference of each variable between each country. The data presented in the article was collected from 969 university students in Malaysia, Indonesia, and Thailand. After eliminated data which present missing or erroneous values (outliers), there are 355 males, and 614 females, with an average age of 20.47 years and a standard deviation of 1.87. Respondents were recruited by simple random sampling where undergraduate students were participated voluntarily, regardless of their gender and socioeconomic status. Respondents who cannot read the questionnaire independently or filled out the questionnaire incompletely and outliers data were excluded. Data collection method in this research is face to face method where the researcher directly met the participant at one time data retrieval. The data collection procedures performed in the same way in three countries. In this data set, variables such as age, gender, and country were included. Specifically, gender was coded 1 for male and 2 for female. The country codes are as follows: 1 for Malaysia, 2 for Indonesia, and 3 for Thailand. Basic information of the sample population can be seen in Tables 1 and 2.

### 2.2 Questionnaires

Questionnaires used in this research were translated based on the Brislin's translation model (1970) [1]. We followed the translation procedures in which a bilingual expert in respective country translated the original version of the scales to Indonesian, Malaysian and Thai language. This is followed by a second bilingual expert who blindly back-translated it to the source language without access to the original language version. After comparing the original version of questionnaires with the back-translated version, terms that are questionable were syntesized, corrected, and retranslated. Subsequently, the questionnaires were back-translated again to see whether the questionable terms were successfully corrected and had an equivalent meaning. Noteworthy, the Malaysian students are presented with a bilingual set of questionnaires (English is widely spoken in Malaysia). CFA method was used to determined valid items for each questionnaires. Items that have factor loading value under 0.3 were ommited and considered not valid. This consideration was based on Hair et al (1998) statement about minimum acceptance of factor loading value [2].

Positive mental health is measured by the 9-item Positive Mental Health Scale [3], and the scale is unidimensional. Items are measured on a 5-point Likert scale ranging from 1 = Strongly Disagree to 5 = Strongly Agree. Reliability analysis for the scale was conducted and the Cronbach Alpha coefficient is 0.88.

Subjective happiness is measured by the 3-item Subjective Happiness Scale, [4] and the scale is unidimensional. Items are measured on a 6-point Likert scale ranging from 1 = Strongly Disagree to 6 = Strongly Agree. Reliability analysis for the scale is good with the Cronbach Alpha coefficient value is 0.79.

Forgiveness is measured by the 12-item Forgiveness Questionnaire [7] which has three dimensions. The dimensions are blockage, circumstances, and forgiveness vs revenge. Items of the scale are rated on a 5-point Likert scale ranging from 1 = Strongly Disagree to 5 = Strongly Agree. Reliability analysis was conducted using SPSS with the Cronbach Alpha coefficient value is 0.84. 10 items from the original questionnaire were ommited based on factor loading value result consideration. Items with a value under 0.3 were ommited leaving the questionnaire with only 12 items from originaly 22 items. Items for blockage dimension were items number 1–3, items for circumstances dimension were items number 4–8, and items for forgiveness vs revenge were items number 9–12.

The fourth variable is humility which was measured by the 2-items Brief State Humility Scale [5]. The scale is unidimensional and the items are measured on a 7-point Likert scale ranging from 1 = Strongly Disagree to 7 = Strongly Agree. As for reliability, analysis was done using SPSS with the Cronbach Alpha coefficient obtained is 0.80. 1 item from the original questionnaire were ommited based on factor loading value result consideration. Items with a value under 0.3 were ommited leaving the questionnaire with only 2 items from originaly 6 items.

Finally, information literacy self-efficacy was measured by the 6-items Information Literacy Self-Efficacy Scale [6]. The scale has three dimensions, and items were measured on a 5-point Likert scale ranging from 1 = Strongly Disagree to 5 = Strongly Agree. The dimensions are basic information literacy, middle information literacy, and high information literacy. Reliability analysis was performed and the Cronbach Alpha coefficient value found was 0.77. 11 items from the original questionnaire were ommited based on factor loading value result consideration. Items with a value under 0.3 were ommited leaving the questionnaire with only 6 items from originaly 17 items. Items basic information literacy were items number 1-3, items for middle information literacy were items number 5-6, amd items for high information literacy were items number 4.

### 2.3 Analyses result

The results of the descriptive statistics (Mean and SD) of the total scores of all the variables in the questionnaires are presented in Table 2. In order to describe the data in an exploratory way, the correlation between the data was computed. The results of the correlational analyses can be seen in Table 4. The influence of subjective happiness, forgiveness, humility, and information literacy self-efficacy on mental health are ascertained through the multilinear regression analysis and the results are shown in Table 5. Finally, to discriminate the differences between each country in terms of each of the variables, we conducted a Kruskal-Wallis analysis and the results are in Table 6.

## Declaration of Competing Interest

Hereby, the authors state that they have no competing financial interests or personal relationships that could influence the work reported in this article.

## Appendix Supplementary materials

Image, application 1

Image, application 2

Image, application 3

Image, application 4
