# Supplementary material for: Dataset on positive mental health of Indonesian, Malaysian, and Thailand university students
Source: Data Brief. 2020 Sep 14;32:106314. doi: 10.1016/j.dib.2020.106314 (PMC7509368; doi:10.1016/j.dib.2020.106314)
Supplement: Supplementary file 2 [file mmc2.doc]

**QUESTIONAIRE**

**MENTAL HEALTH AND HAPPINESS**

This research is a psychological study aimed at identifying the relationship between mental health with happiness and several other variables. This is a collaborative research between Tutut Chusniyah, Dedi Kuswandi, and Gebi Angelina Zahra (State University of Malang), with Jas Laile Suzana Binti Jaafar (University of Malaya) and Apitchaya Chaiwutikornwanich (Chulalongkorn University). We expect your willingness to fill out the questionnaire by answering each question honestly and spontaneously. All information obtained through this questionnaire is confidential however only questionnaires with complete responses will be processed. Therefore, please complete every sections of this questionnaire. If you have any further questions, please send it to tututchusniyah@gmail.com. Your cooperation is very much appreciated, thank you.

Best Regards,

Tutut Chusniyah

Dedi Kuswandi

Gebi Angelina Zahra

(Universitas Negeri Malang)

Jas Laile Suzana Binti Jaafar

(University of Malaya)

Apitchaya Chaiwutikornwanich

(Chulalongkorn University)

**INFORMED CONSENT**

I, the undersigned below:

| Name | : |  |
| --- | --- | --- |
| Age | : |  |
| Sex | : |  |
| Phone number | : |  |
| I declare that | : |  |
| I have obtained an explanation of the objectives, benefits, procedures, and risks and got a satisfying answer to the question I asked for a study entitled "Positive Mental Health of Indonesian, Malaysian, and Thailand University Students Facing Industrial Revolution 4.0".  I hereby declare that I am willing to include myself voluntarily in this research. I understand that I am allowed to resign from this research. | | |

| Researcher | Study Participants | Witness |
| --- | --- | --- |
|  |  |  |
| (Tutut Chusniyah) | ( ) | ( ) |

**PART A: PARTICIPANT’S IDENTITY**

Please fill this column

| Age | : |  |
| --- | --- | --- |
| Sex | : | Male/Female |
| University Affiliation | : |  |
| Religion | : |  |
| Ethnic/Tribe | : |  |

**PART B: HAPPINESS SCALE**

Instruction: For each of the statements below, cross the numbers that you think are the most appropriate in explaining yourself.

1. In general, I consider myself:

not a very happy person ---------------------------------------------------- a very happy person

1 2 3 4 5 6

1. Compared to most of my peers, I consider myself:

less happy ---------------------------------------------------------------------------- more happy

1 2 3 4 5 6

1. Some people are generally very happy. They enjoy life regardless of what is going on, getting the most out of everything. To what extent does this characterization describe you? not at all ------------------------------------------------ ------------------------------ a great deal

1 2 3 4 5 6

1. Some people are generally not very happy. Although they are not depressed, they never seem as happy as they might be. To what extend does this characterization describe you?

not at all ------------------------------------------------------------------------------ a great deal

1 2 3 4 5 6

**PART C: FORGIVENESS SCALE**

This section contains 27 statements about forgiveness. For each of the following statements, circle the number indicating the extent to which you agree or disagree with the statement, based on: value 1 = strongly disagree, to value 5 = strongly agree. Please answer honestly.

| **NO** | **STATEMENTS** |  | | | | |
| --- | --- | --- | --- | --- | --- | --- |
| 1 | I cannot forgive if the offender has not apologized | 1 | 2 | 3 | 4 | 5 |
| 2 | I cannot forgive if the consequences of harm have not cancelled | 1 | 2 | 3 | 4 | 5 |
| 3 | I cannot forgive if the consequence of harm are serious | 1 | 2 | 3 | 4 | 5 |
| 4 | I cannot forgive if the offender has not begged for forgiveness | 1 | 2 | 3 | 4 | 5 |
| 5 | I always apply the lex Talionis | 1 | 2 | 3 | 4 | 5 |
| 6 | The way I consider the world has brought me to never forgive | 1 | 2 | 3 | 4 | 5 |
| 7 | I cannot forgive even if the offender has apologized | 1 | 2 | 3 | 4 | 5 |
| 8 | cannot forgive even if my family or friends have invited me to do so | 1 | 2 | 3 | 4 | 5 |
| 9 | I cannot forgive even if a religious figure has asked me to do so | 1 | 2 | 3 | 4 | 5 |
| 10 | I can truly forgive if the little harm has been done | 1 | 2 | 3 | 4 | 5 |
| 11 | I can truly forgive only if I have been able to take revenge on the offender | 1 | 2 | 3 | 4 | 5 |
| 12 | I cannot forgive even if the consequences of harm have canceled | 1 | 2 | 3 | 4 | 5 |
| 13 | I cannot forgive even if the consequences of harm are minimal | 1 | 2 | 3 | 4 | 5 |
| 14 | My personal philosophy does not bring me to forgive | 1 | 2 | 3 | 4 | 5 |
| 15 | I forgive easily even when I feel bad | 1 | 2 | 3 | 4 | 5 |
| 16 | My personal philosophy or my faith has brought me to always to forgive | 1 | 2 | 3 | 4 | 5 |
| 17 | I think it is better to forgive than to seek revenge | 1 | 2 | 3 | 4 | 5 |
| 18 | I can forgive easily even when the offender has not begged for | 1 | 2 | 3 | 4 | 5 |
| 19 | I can forgive easily even if the consequences of harm have not canceled | 1 | 2 | 3 | 4 | 5 |
| 20 | I can truly forgive even if the consequences are serious | 1 | 2 | 3 | 4 | 5 |
| 21 | I can easily forgive even when the offender has not apologized | 1 | 2 | 3 | 4 | 5 |
| 22 | I forgive more easily if the offender has apologized | 1 | 2 | 3 | 4 | 5 |
| 23 | I forgive more easily if the offender has begged for forgiveness | 1 | 2 | 3 | 4 | 5 |
| 24 | I feel it is easier to forgive once the consequences of harm have canceled | 1 | 2 | 3 | 4 | 5 |
| 25 | I forgive more easily when I feel good | 1 | 2 | 3 | 4 | 5 |
| 26 | I feel it is easier to forgive when my family or friends have invited me to do | 1 | 2 | 3 | 4 | 5 |
| 27 | I forgive more easily if a religious figure has invited me to do so | 1 | 2 | 3 | 4 | 5 |

**PART D: POSITIVE MENTAL HEALTH SCALE**

This section contains 9 statements about positive mental health. For each of the following statements, circle the numbers to indicate your agreement or disagreement with the statement, based on: value 1 = strongly disagree, to value 5 = strongly agree. Please answer honestly.

| **NO** | **STATEMENTS** |  | | | | |
| --- | --- | --- | --- | --- | --- | --- |
| 1 | I am often carefree and in good spirits | 1 | 2 | 3 | 4 | 5 |
| 2 | I enjoy my life | 1 | 2 | 3 | 4 | 5 |
| 3 | All in all, I am satisfied with my life | 1 | 2 | 3 | 4 | 5 |
| 4 | In general, I am confident | 1 | 2 | 3 | 4 | 5 |
| 5 | I manage well to fulfill my needs | 1 | 2 | 3 | 4 | 5 |
| 6 | I am in good physical and emotional condition | 1 | 2 | 3 | 4 | 5 |
| 7 | I feel that I am actually well equipped to deal with life and its difficulties | 1 | 2 | 3 | 4 | 5 |
| 8 | Much of what I do brings me joy | 1 | 2 | 3 | 4 | 5 |
| 9 | I am a calm, balanced human being. | 1 | 2 | 3 | 4 | 5 |

**PART E: HUMILITY SCALE**

This section contains 6 statements about humility. For each of the following statements, circle the numbers to indicate your agreement or disagreement with the statement, based on: value 1 = strongly disagree, to value 7 = strongly agree. Please answer honestly.

| **NO** | **STATEMENTS** |  | | | | | | |
| --- | --- | --- | --- | --- | --- | --- | --- | --- |
| 1 | I feel that, overall, I am no better or worse than the average person | 1 | 2 | 3 | 4 | 5 | 6 | 7 |
| 2 | I feel that I have both many strengths and flaws. | 1 | 2 | 3 | 4 | 5 | 6 | 7 |
| 3 | I feel that I do not deserve more respect than other people. | 1 | 2 | 3 | 4 | 5 | 6 | 7 |
| 4* | To be completely honest, I feel that I am better than most people. | 1 | 2 | 3 | 4 | 5 | 6 | 7 |
| 5* | I feel that I deserve more respect than everyone else. | 1 | 2 | 3 | 4 | 5 | 6 | 7 |
| 6* | I feel that I do not have very many weaknesses. | 1 | 2 | 3 | 4 | 5 | 6 | 7 |

**PART F: LITERACY INFORMATION SELF-EFFICACY SCALE**

This section contains 17 statements about literacy information self-efficacy. For each of the following statements, circle the numbers to indicate your agreement or disagreement with the statement, based on: value 1 = strongly disagree, to value 5 = strongly agree. Please answer honestly.

**I feel confident and competent to:**

| **NO** | **STATEMENTS** |  | | | | |
| --- | --- | --- | --- | --- | --- | --- |
| 1. | Define the information I need | 1 | 2 | 3 | 4 | 5 |
| 6 | Use different kinds of print sources (i.e. books, periodicals, encyclopedias, chronologies, etc.) | 1 | 2 | 3 | 4 | 5 |
| 7 | Use electronic information sources | 1 | 2 | 3 | 4 | 5 |
| 8 | Locate information sources in the library | 1 | 2 | 3 | 4 | 5 |
| 9 | Use library catalogue | 1 | 2 | 3 | 4 | 5 |
| 10 | Locate resources in the library using the library catalogue | 1 | 2 | 3 | 4 | 5 |
| 15 | Select information most appropriate to the information need | 1 | 2 | 3 | 4 | 5 |
| 18 | Synthesize newly gathered information with previous information | 1 | 2 | 3 | 4 | 5 |
| 19 | Interpret the visual information (i.e. graphs, tables, diagrams) | 1 | 2 | 3 | 4 | 5 |
| 20 | Write a research paper | 1 | 2 | 3 | 4 | 5 |
| 21 | Determine the content and form the parts (introduction, conclusion) of a presentation (written, oral) | 1 | 2 | 3 | 4 | 5 |
| 22 | Prepare a bibliography | 1 | 2 | 3 | 4 | 5 |
| 23 | Create bibliographic records and organize the bibliography | 1 | 2 | 3 | 4 | 5 |
| 24 | Create bibliographic records for different kinds of materials (i.e. books, articles, web pages) | 1 | 2 | 3 | 4 | 5 |
| 25 | Make citations and use quotations within the text | 1 | 2 | 3 | 4 | 5 |
| 27 | Learn from my information problem solving experience and improve my information literacy skill | 1 | 2 | 3 | 4 | 5 |
| 28 | Criticize the quality of my information seeking process and its products | 1 | 2 | 3 | 4 | 5 |

**THANK YOU**
